# How axon and dendrite branching are guided by time, energy, and spatial constraints

**DOI:** 10.1038/s41598-022-24813-2

**Published:** 2022-12-02

**Authors:** Paheli Desai-Chowdhry, Alexander B. Brummer, Van M. Savage

**Affiliations:** 1 Department of Computational Medicine, University of California Los Angeles, BOX 951766, Room 5303 Life Sciences, Los Angeles, CA 90095-1766 USA; 2 Department of Ecology and Evolutionary Biology, University of California Los Angeles, Los Angeles, CA USA; 3 Santa Fe Institute, Santa Fe, NM USA; 4 Present Address: Department of Physics and Astronomy, College of Charleston, Charleston, SC USA

**Keywords:** Computational biophysics, Neuroscience, Cellular neuroscience, Computational neuroscience, Applied mathematics

## Abstract

Neurons are connected by complex branching processes—axons and dendrites—that process information for organisms to respond to their environment. Classifying neurons according to differences in structure or function is a fundamental part of neuroscience. Here, by constructing biophysical theory and testing against empirical measures of branching structure, we develop a general model that establishes a correspondence between neuron structure and function as mediated by principles such as time or power minimization for information processing as well as spatial constraints for forming connections. We test our predictions for radius scale factors against those extracted from neuronal images, measured for species that range from insects to whales, including data from light and electron microscopy studies. Notably, our findings reveal that the branching of axons and peripheral nervous system neurons is mainly determined by time minimization, while dendritic branching is determined by power minimization. Our model also predicts a quarter-power scaling relationship between conduction time delay and body size.

## Introduction

Neurons are fundamental structural units of information processing and communication in animals. They are made up of a centralized cell body, called the soma, and two types of extending processes, axons and dendrites. These processes transfer information between cells in the form of electrical and chemical signals. Axons generally conduct signals from the cell body to the synapses, where they connect with the dendrites of other neurons. These dendrites generally conduct signals from the synapse to the cell body. The processes form synaptic connections with one another in complex patterns. Different types of cells exhibit diverse morphological forms—some neurons have no axons or dendrites, while some have long axon processes that extend over meters, and others have vast dendritic trees that branch extensively to fill two- or three-dimensional space, corresponding to the mathematical and modeling concept known as space-filling^1^.

Seminal studies in neuroscience characterized morphological differences across cell types. For instance, Santiago Ramón y Cajal’s “Histology of the Nervous System of Man and Vertebrates” is considered to be the founding document of neurobiology^2^, consisting of detailed drawings and comparative descriptive analysis of neuron morphology across different cell types and species^3^. Modern techniques and devices have allowed for more precise quantitative measurements at the single-cell level. For example, recent work has established quantitative morphological distinctions across different cell types, focusing on quantities such as mean dendritic length, total dendritic length, and number of branching points^4,5^.

As vast as the structural diversity is, there is an even greater diversity of functional properties^1^. Within sensory, motor, and interneurons, there are different types of neurotransmitters and receptors that affect the nature of signal processing^6^. A major future goal of neuron cell-type classification is to establish a correspondence between morphological and functional properties^2^. Here, we seek to address the question of how structural properties relate to neuron function and whether there are evolutionary driving forces that dictate how morphology is optimized by biological principles or pressures.

A promising approach to the relationship between neuron structure and function is biological scaling theory, as it has previously been applied to understand patterns in the branching structures of biological resource distribution networks. Generally, a biological property Y scales with body mass M as $Y = Y_0 M^{b}$, where $Y_0$ is a proportionality constant and b is a scaling exponent^7^. An example is metabolic rate scaling with body mass to the power 3/4, a result known as Kleiber’s Law^8^.

West, Brown, and Enquist (WBE) proposed that Kleiber’s law and other biological scaling laws arise because biological organisms are sustained by resource distribution branching networks that are optimized to supply all parts of the body^7^. Past work on cardiovascular networks has employed WBE theory to derive scaling laws for the vessel radius and length as a result of minimizing power loss for fluid flow along with space filling in order to fuel whole organism metabolism^9^. Moreover, previous results have shown a quarter-power allometric scaling relationship between cell size and body size in a range of cell types in mammals, including neurons^10^.

Single neuron cells have centralized cell bodies that are analogous to the heart and branching processes that are analogous to blood vessels. Consequently, we propose that a similar approach based on optimizing organismal function subject to biophysical constraints may be fruitful for attempting to predict and understand the branching structures of axons and dendrites. As such, we consider biophysical properties of neurons that might play an important role in governing structure, and we use empirical imaging data to guide our evaluation of the relative importance of different functions.

One important evolutionary function of neuronal networks is the transferring of large amounts of information between brain regions in a short amount of time^11^. At the individual cell level, the varied morphological forms observed for neurons are various adaptations to basic principles such as limiting signal time delay^3^. Thus, it is important to consider conduction time as a key design principle that governs neuronal branching structures.

Indeed, foundational work by Cuntz et al. has used graph theory to quantify and study how connections among axons and dendrites determine conduction time delay. This approach focuses on the tradeoff between wiring costs and conduction time, represented as path length^12^. The results formalize the laws set forth by Ramón y Cajal, leading to a graph-theoretical algorithm that generates synthetic and biologically accurate axonal and dendritic trees^13^.

Although this formalism is deeply insightful and very successful at explaining neuron structures, two key aspects are absent for optimizing Ramón y Cajal’s laws: the diameter of axonal and dendritic fibers is not incorporated, and the principle of conservation of space, as set forth by Ramón y Cajal, is missing. Because axon and dendrite radius relates to resistance to the flow of electrical current, it has a profound effect on signaling speed and conduction time. The radius is thus a key structural feature governing the function of neurons. Moreover, space-filling principles constrain the possible connections, branching, and network structure of neurons. Consequently, in this paper we take a similar approach to Cuntz et al.^12,14^ except we now incorporate the dependence of conduction time on fiber radius and myelination (insulation that surrounds the fiber and facilitates signal transduction^6^), using principles set forth by Hodgkin and Rushton^15,16^, along with the principle of space-filling.

A complicating factor that creates a tradeoff is that as the speed of information processing increases, energy loss due to dissipation also increases^11^. Indeed, signaling in the brain consumes a substantial amount of energy^17^, suggesting that energy expenditure is another important factor constraining neuron structure. Previous work has shown that the relationship between metabolic rate and conduction time plays an important role in determining axon function in species across scales of body size^18^. This leads to the WBE framework, which relies on the assumption that resource distribution networks are optimized such that the energy used to transport resources is minimized^7^. This framework is applied to cardiovascular networks by minimizing power lost to dissipation in small vessels, leading to the derivation of a power law (also known as Murray’s law), which states that the radius scales with an exponent of 3 in branching blood vessels^9^.

Importantly, Wilfred Rall derived a similar power law for neurons. By using the assumption that the charge is conserved at branching junctions, the diameter of daughter branches and parent branches can be related by an exponent of 3/2^19^. Rall found that this power law holds for motoneurons but not for other cell types. Because of this seminal work, many subsequent theoretical and experimental studies on the scaling of neuron branching have used Rall’s law as a baseline for quantifying variation in scaling exponents by calculating departures and differences from the 3/2-value of the scaling exponent for Rall’s law^20,21^. Although there are many functional differences between cardiovascular networks and neurons, the presence of these analogous scaling laws suggests that the mathematical framework applied to branching blood vessels might be useful to apply to neurons. While energy efficiency is the primary constraining factor considered in studies of cardiovascular systems, neurons differ in that information processing and signalling is a key consideration. We use the objective function approach from biological scaling theory for cardiovascular systems as a basis and use it to construct new models and functions that incorporate characteristic properties of neurons across their vast diversity of cell types, morphologies, and physiological functions. Applying this lens to look at neurons will expand upon existing frameworks, and comparative studies might help capture more nuances in other biological systems as well.

In this paper, we show that much of the variation around Rall’s law can be explained and predicted using our approach-varying the relative importance and weighting of time versus energy and the associated biological and physical constraints to consider a host of functions that can be optimized to derive predictions for diverse morphological quantities. That is, building on biological and physical principles that constrain electrophysiological signaling and information processing in neurons, we construct a general model that predicts a suite of neuron morphologies based on which biological or physical principles are under the strongest selection. Our model includes both conduction time and energy efficiency while also incorporating additional factors such as the material costs and space-filling^3^. We make theoretical predictions for how branch radius changes across branching generation for both axons and dendrites. We compare these predictions to our empirically measured data to make conclusions about the functional basis for morphological differences observed across cell types. We also use this model to predict how conduction time delay in neurons changes with neuron size, another of our predictions that is supported by empirical data. We use data collected from light and electron microscopy. These data collected by different methods yield extremely consistent results, strengthening support for our general model as a basis to predict neuron morphology.

## Theory

### Model

Because conduction time delay and power usage are fundamental and costly for information processing in neurons, we develop a mathematical objective function to jointly minimize time and power that is subject to constraints^22^. By deriving the minimization conditions, we predict how biological principles and constraints govern neuronal structure, as achieved via evolutionary pressures and developmental processes that shape branching networks and materials, such as myelination. Our work differs substantially from work on the cardiovascular system that does not consider effects of conduction time delay, as the physiology and function of neurons conducting currents differ in essential ways from blood vessels transporting blood. As a consequence, we also obtain different scaling relations than for the cardiovascular system. Here, we obtain a suite of predictions based on the details of the particular type of neuron such as functional differences and myelination.

Equation (1) is a general form of our objective function. The biophysical constraints are represented as functions and added to the expressions to be minimized, allowing us to use the method of undetermined Lagrange multipliers to optimize this overall objective function.

1 $$\begin{aligned} F = \alpha P_{TOT} + (1-\alpha ) T_{TOT} + \sum _i^N \uplambda _i f_i(r_k, l_k, k, n, m_c, d) \end{aligned}$$

**Figure 1:**
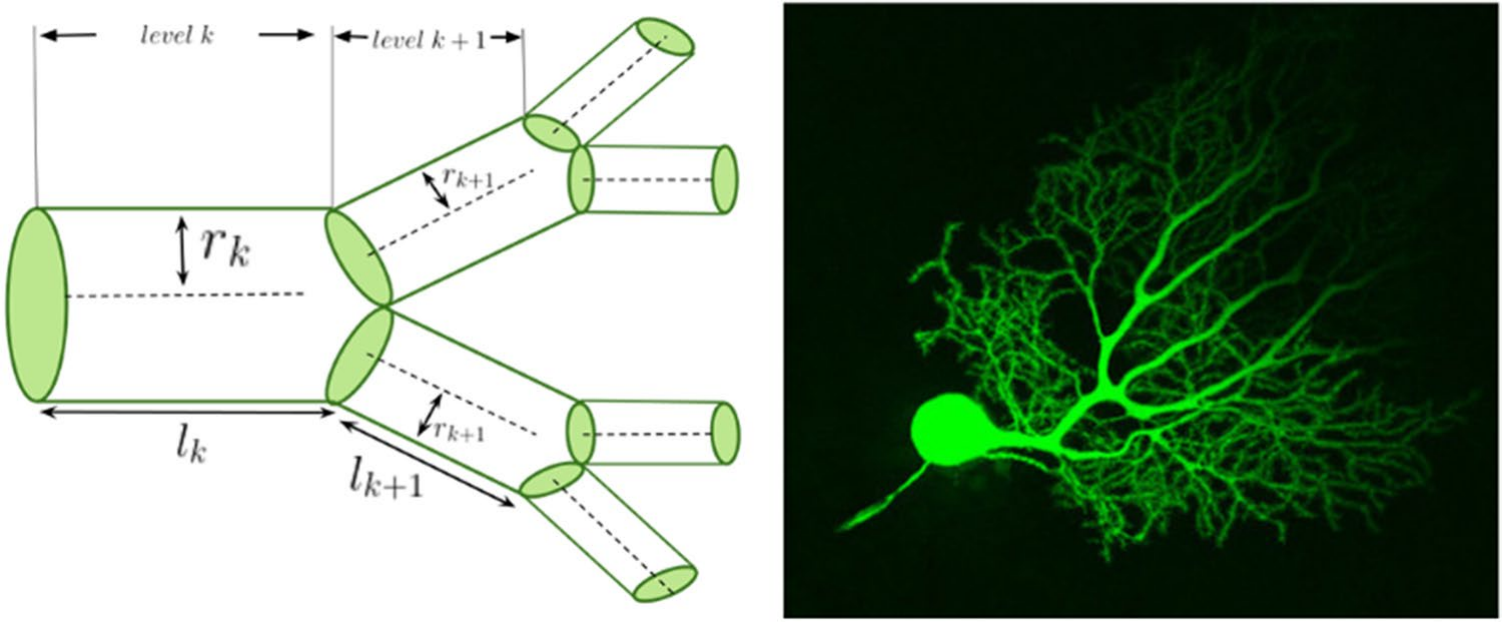
A hierarchical branching network. A visual depiction of the successive branching levels of a network and the quantities of interest alongside an image of a mouse cerebellar Purkinje neuron and its dendritic branching structure. This image was obtained using confocal microscopy and Lucifer yellow fluorescent dye. We have cropped this image available on CellImageLibrary.Org, distributed by Maryann Martone, Diana Price, and Andrea Thor^23^.

Here, $P_{TOT}$ is the power lost due to dissipation and $T_{TOT}$ is the time delay for a signal travelling across the network. The parameter $\alpha$ can be varied to consider the tradeoff between these two principles in governing the structure of different cell types. The functions $f_i$ represent biophysical constraint functions that generically depend on the branch radius $r_k$ and the branch length $l_k$, where *k* is the branching generation of the network (with 0 being the trunk and *N* being the tips). This function also depends on the total number of branching levels *N* of a neuron process, the branching ratio *n*, the mass of the cell $m_c$, and the dimension *d* of space into which the neuron processes project. The branching ratio, *n*, is equal to 2 for a bifurcating network, though it may vary in general. We use optimization methods to calculate scaling relationships between the radius of successive branches, $\frac{r_{k+1}}{r_k}$, as shown in Fig. 1.

In Eq. (1), the first term is the power loss due to dissipation, given by $P_{TOT} = \sum _{k=0}^N \frac{l_k}{r_k^2 n^k}$. For a neuronal network, we define the power loss by the equation, $P = I_0^2 R_{net}$, where $I_0$ is the ionic current and $R_{net}$ is the resistance to current flow in the network. Because we are focusing on average, large scale quantities across the full extent of the neuron and need to consider a coarse-grained average of signal propagation, we can reasonably approximate axons and dendrites as wires through which current flows and encounters resistance from the neuron fiber. The resistance is given by $R_k = \frac{\rho l_k }{A_k}$, where $A_k$ is the cross sectional area of the wire, and $l_k$ is the length of the segment at that level. The parameter $\rho$ is the intrinsic resistivity of the axon or dendrite, and we assume that $\rho$ is constant, meaning that the material is uniform^1^. Approximating axons and dendrites as cylinders, the cross sectional area is $\pi r_k^2$ for level k, and the resistance is $R_k = \frac{\rho l_k }{\pi r_k^2}$. Following standard practice, we have absorbed all physical constants into the Lagrange constants, and the magnitude of these terms do not affect the theoretical predictions.

The second term represents conduction time delay, $T_{TOT} = \sum _{k=0}^N \frac{l_k}{r_k^{\frac{1}{2}+ \epsilon }}$, and arises because the average velocity of a signal along a single branch is $\bar{v} = l_k/t_k$, where $t_k$ is the time delay. We can solve this expression for $t_k$ and sum $\frac{1}{\bar{v}}$ over the length of each branch^24^. At each generation, we consider a single branch to denote the total path length of a signal, and we calculate the total conduction time delay by summing the time delays for single branches across all *N* generations. The parameter $\epsilon$ describes the degree of myelination. Previous work has shown that the conduction speed is proportional to the square root of the diameter for an unmyelinated fiber^15^, and directly proportional to the diameter for a myelinated fiber^16^. Thus, an $\epsilon$ value of 0 corresponds to an unmyelinated fiber, and a value of $\frac{1}{2}$ corresponds to a myelinated fiber.

Notably, we include linear terms for $P_{TOT}$ and $T_{TOT}$, consistent with approaches for other systems in physics and economics. Because our quantities are always positive based on their biological interpretation, minimizing these terms and the overall functions effectively minimizes all monotonic transformations such as positive powers or logarithms of the function. Consequently, our predicted minima and scaling ratios actually hold for a broad range of functional forms (in the same way that maximum likelihood and maximum entropy calculations minimize the logarithm of the function to also find the minimum of the linear form). In addition, many complicated nonlinear functions behave linearly near critical points and linearity is the simplest form in lieu of additional information, providing additional reasons for its use as a starting point. Future studies may expand on this baseline model to consider other mathematical forms if sound biological justification is provided for their consideration.

With this form of the objective funciton, we can switch between models that optimize either conduction time or power usage by varying $\alpha$ between 0 and 1, corresponding to the following two equations.

2 $$\begin{aligned} T= & {} \sum _{k=0}^N \frac{l_k}{r_k^{\frac{1}{2}+ \epsilon }} + \uplambda \sum _{k=0}^N n^k r_k^2 l_k + \uplambda _m m_c + \sum _{k=0}^N \uplambda _k n^kl^d \end{aligned}$$

3 $$\begin{aligned} P= & {} \sum _{k=0}^N \frac{l_k}{r_k^2 n^k} + \uplambda \sum _{k=0}^N n^k r_k^2 l_k + \uplambda _m m_c + \sum _{k=0}^N \uplambda _k n^kl^d \end{aligned}$$

In these two equations, the governing optimization principle (first term) is constrained by brain region volume (second term), neuron size (third term), and space-filling (fourth term). These quantities are held constant during the optimization. We can define the total volume as $\sum _{k=0}^N n^k \pi r_k^2 l_k$, based on the assumption that the projections are cylindrical and branches are symmetric. We absorb the constant $\pi$ into the Lagrange multiplier $\uplambda$. The term that describes size, $m_c$, is the mass of the cell. The last constraint comes from the fact that a resource distribution network must extend and branch out to reach as much of the space it fills as possible, whether it is a 2-dimensional plane or 3-dimensional volume. Each branch in a given level of the network reaches a fixed volume of cells in the space it fills, called the service volume, and the total service volume at each level is preserved. The service volumes vary in proportion to $l_k^d$, so the total volume is proportional to the service volumes summed across each of the branches at a given generation k, $n^kl_k^d$^9^. Thus, this quantity is held constant as a constraint function at each level k in the *N* generations. We assume that the branching ratio is constant, so the number of vessels at level k is $n^k$.

The predictions that arise from these two functions will correspond to different cell types and processes based on differences in signaling properties. We hypothesize that the principle of power loss due to dissipation will be favored in determining the structure of processes that rely on passive electronic spread, such as dendrites, while the principle of conduction time delay will be favored in determining the structure of processes that are electrically active and conduct action potentials, such as axons. The power term describes the attenuation of a signal depending on the internal resistivity of the process. For processes that conduct action potentials, the time delay term is a more useful descriptor, as it accounts for signaling not limited to passive attenuation and considers differences observed in myelinated and unmyelinated fibers. Through the tradeoff between these principles, our general model accounts for both types of signaling.

Furthermore, we note that the energetic cost of maintaining the resting membrane potential is already captured in the constraint for the total network volume. This is crucial when considering energy consumption in neurons because a large component is involved in maintaining the resting membrane potential that goes beyond energy loss to dissipation^17^. This resting membrane potential depends on the energy required by the sodium-potassium pump, which increases with surface area of the neuron^17^. Increasing surface area corresponds to an increase in volume if we assume that myelination has minimal affects on the total surface area (see “Discussion”), so that energy consumption is a per-volume quantity^18^.

Crucially and somewhat surprisingly, the space-filling constraint does not change any of our predictions for radius scaling ratios. However, space-filling does play a crucial role in determining how neuron size scales with body size across species, as well as the predictions for length scaling ratios, $\frac{l_{k+1}}{l_k}$, as shown in Supplementary *Information: Text  S1. Notably, we do not compare these predictions to empirical length scaling ratios here because that would likely require the introduction and usage of an alternative labeling scheme like the Horton–Strahler method as opposed to the generational labeling scheme for branch radius throughout the rest of this paper (see “Discussion”). As such, we leave that construction and analysis to future work in which we will delve deeper into asymmetric branching and alternative labeling schemes.

In “Allometry calculation”, we show that minimizing power subject to a conduction time delay constraint leads to a $\frac{1}{4}$-power scaling between conduction time delay and neuron size. This objective function can be described by the following equation:

4 $$\begin{aligned} P^* = \sum _{k=0}^N \frac{l_k}{r_k^2 n^k} + \uplambda \sum _{k=0}^N \frac{l_k}{r_k^{\frac{1}{2} + \epsilon }} + \uplambda _m m_c + \sum _{k=0}^N \uplambda _k n^kl^d \end{aligned}$$

Equations (2), (3), and (4) are all specific cases of the more general Eq. (1), with varying values of $\alpha$ as well as choice of constraint functions.

### Scaling ratio calculation

We use the method of Lagrange multipliers to solve for the values of the scaling ratios for radius, $\frac{r_{k+1}}{r_k}$ that minimize the objective function. Below, we show a sample calculation of the method of Lagrange multipliers for the case of power minimization (Eq. 3). A more detailed calculation can be found in Supplementary Information: Text  S1.

We will first minimize P by differentiating with respect to radius at an arbitrary level *k* and setting the result equal to 0.

5 $$\begin{aligned} \frac{\partial P}{\partial r_k} = \frac{-2l_k}{n^k r_k^3} + 2 \uplambda n^k r_k l_k = 0 \end{aligned}$$

Solving for the Lagrange multiplier, we have

6 $$\begin{aligned} \uplambda = \frac{1}{n^{2k}r_k^{4}} \end{aligned}$$

Using the fact that the Lagrange multiplier is a constant and thus the denominator must be constant across levels, we can solve for the scaling ratio

7 $$\begin{aligned} \frac{r_{k+1}}{r_k} = n^{-1/2} \end{aligned}$$

This method is used to solve for the scaling ratios for radius for the other cases and compared to empirical results. These findings are summarized in Table 1 in “Results” section.

To ease comparison with previous work, we first note that the convention in neuroscience is to quantify changes in branching radius by using the following equation that relates the diameter of a parent branch to the two daughter branches:

8 $$\begin{aligned} d_0^{\eta } = d_1^{\eta } + d_2^{\eta } \end{aligned}$$

We next note that much existing work presents results as a measure of departure from the baseline of Rall’s law, corresponding to $\eta = \frac{3}{2}$^19^.

Conveniently, for symmetrically branching networks, there is a simple correspondence between this conventional formulation, focusing on scaling exponents relative to Rall’s law, and our model, focusing on radius scaling ratios

9 $$\begin{aligned} \frac{r_{k+1}}{r_k} = n^{-1/\eta } \end{aligned}$$

For example, Rall’s law, $\eta = \frac{3}{2}$, corresponds to the radius scaling ratio $\frac{r_{k+1}}{r_k} = n^{-2/3}$ in our framework. This equation allows for a simple translation for us to validate our general model’s various predictions due to the tradeoff of different functional principles in the context of existing results in the neuroscience literature.

### Allometry calculation

We now use the objective function P* (Eq. 4), to derive a functional scaling relationship between conduction time delay and body mass. Here, we consider conduction time delay as a constraint, focusing on the unmyelinated case ($\epsilon$ = 0), and considering the case of 3-dimensional space filling (*d* = 3).

We begin by taking the derivative of P* with respect to radius and length and then setting these derivatives equal to zero to solve for the multipliers $\uplambda$ and $\uplambda _k$, respectively, at the stationary point. Substituting the expression for $\uplambda _k$ back into the original expression for $P^*$, we get an expression that simplifies to the original power term that it minimized, $\sum _{k=0}^N \frac{l_k}{r_k^2n^k}$. For simplicity, we replace the power term with *P* and the time delay constraint term with *T* and rearrange. This calculation is shown in detail in Supplementary Information: Text  S2.

Previous results have shown a proportional relationship between $m_c$, the mass of a single cell, and the fourth root of an animal’s body mass, $M^{1/4}$, on average, though specific cell types might vary slightly from this general rule^10^. Thus, we can replace this term and consider a new Lagrange multiplier with the absorbed constant

10 $$\begin{aligned} P^* = 2P+ \uplambda T + \uplambda _M M^{1/4} \end{aligned}$$

We will now take the derivative of this term with respect to M, the mass, and set it equal to 0.

11 $$\begin{aligned} \frac{\partial P^*}{\partial M} = 2\frac{\partial P}{\partial M} + \uplambda \frac{\partial T}{\partial M} + \uplambda _M \frac{\partial M^{1/4}}{\partial M} = 0 \end{aligned}$$

Previous results have shown that the energetic cost, which we have interpreted here as power loss due to dissipation, decreases with increasing body weight of animals at a linear rate on average for both myelinated and unmyelinated fibers, as metabolic rate is a per volume quantity^18^. Thus, we can express $\frac{\partial P}{\partial M}$ generally as a negative constant, $-C$. We can rewrite the above expression as follows

12 $$\begin{aligned} \frac{\partial T}{\partial M} = \frac{- \uplambda _M M^{-3/4} }{4\uplambda } + 2\frac{C}{\uplambda } \end{aligned}$$

Solving and applying the initial condition that T = 0 when M = 0, we have

13 $$\begin{aligned} T = \frac{-\uplambda _M}{\uplambda } M^{1/4} + \frac{2C}{\uplambda } M \end{aligned}$$

Thus, from this equation, we have extracted the scaling relationship—a mixed power law relationship that includes a $\frac{1}{4}$-power law and a linear term with relative weights. Figure 5 shows experimental data that support this theoretical result of the $\frac{1}{4}$-power law.

## Methods

**Figure 2:**
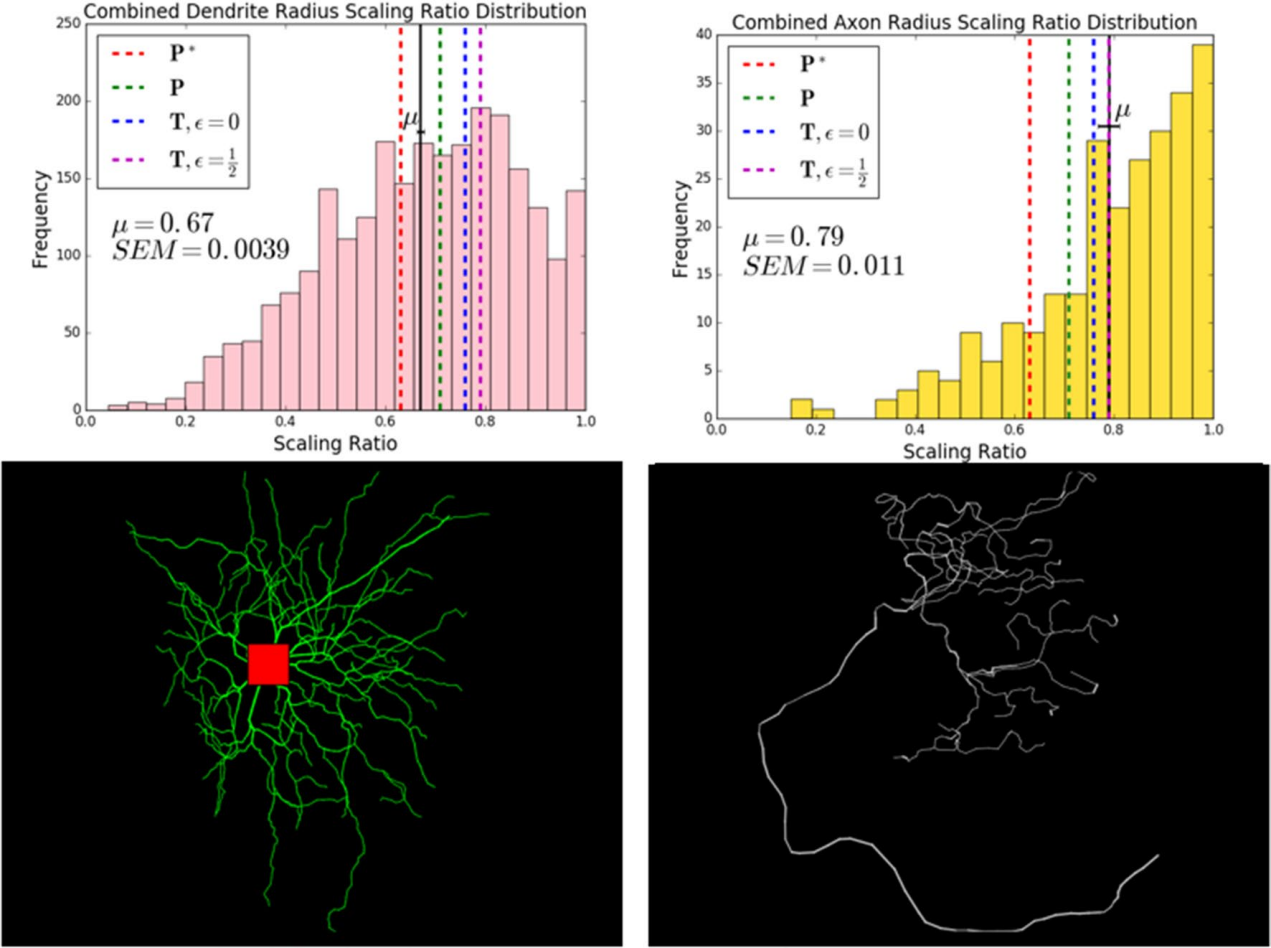
Comparison of dendrite and axon radius scaling ratio distributions, combined. Histograms showing the distributions of radius scaling ratios for axons and dendrites combined from a range of species, brain regions, and cell types available on NeuroMorpho.Org. The mean dendrite scaling ratio is $0.67 \pm 0.004$ and the mean axon scaling ratio is $0.79 \pm 0.01$. In the figure, $\mu$ represents the mean and *SEM* represents the standard error of the mean (SEM). The standard deviations of the distributions are 0.20 for dendrites and 0.17 for axons. The black solid lines denote the mean in the distributions, shown with error bars, and the red, green, blue, and magenta dashed lines represent the theoretical predictions for various objective functions. The closest theoretical predictions for the dendrite scaling ratio mean are the optimal scaling ratios for function $\textit{P}$, minimizing power with fixed volume, $n^{-1/2} \approx 0.71$, and for function $\textit{P*}$, minimizing power with fixed time delay, $n^{-2/3} \approx 0.63$. The closest theoretical predictions for the axon scaling ratio mean are the optimal scaling ratios for function $\textit{T}$, minimizing time delay, the myelinated case with $\epsilon = \frac{1}{2}$, $n^{-1/3} \approx 0.79$, and the unmyelinated case with $\epsilon = 0$, $n^{-2/5} \approx 0.76$. We restricted radius scaling ratio data to values that are less than 1.0. The representative reconstruction images show the characteristic differences in morphology between dendritic and axonal trees. The dendritic tree, shown on the left, is taken from an elephant cerebellar Golgi cell^28^. The axonal tree, with a representative long parent branch, is taken from a mouse touch receptor^53^.

**Figure 3:**
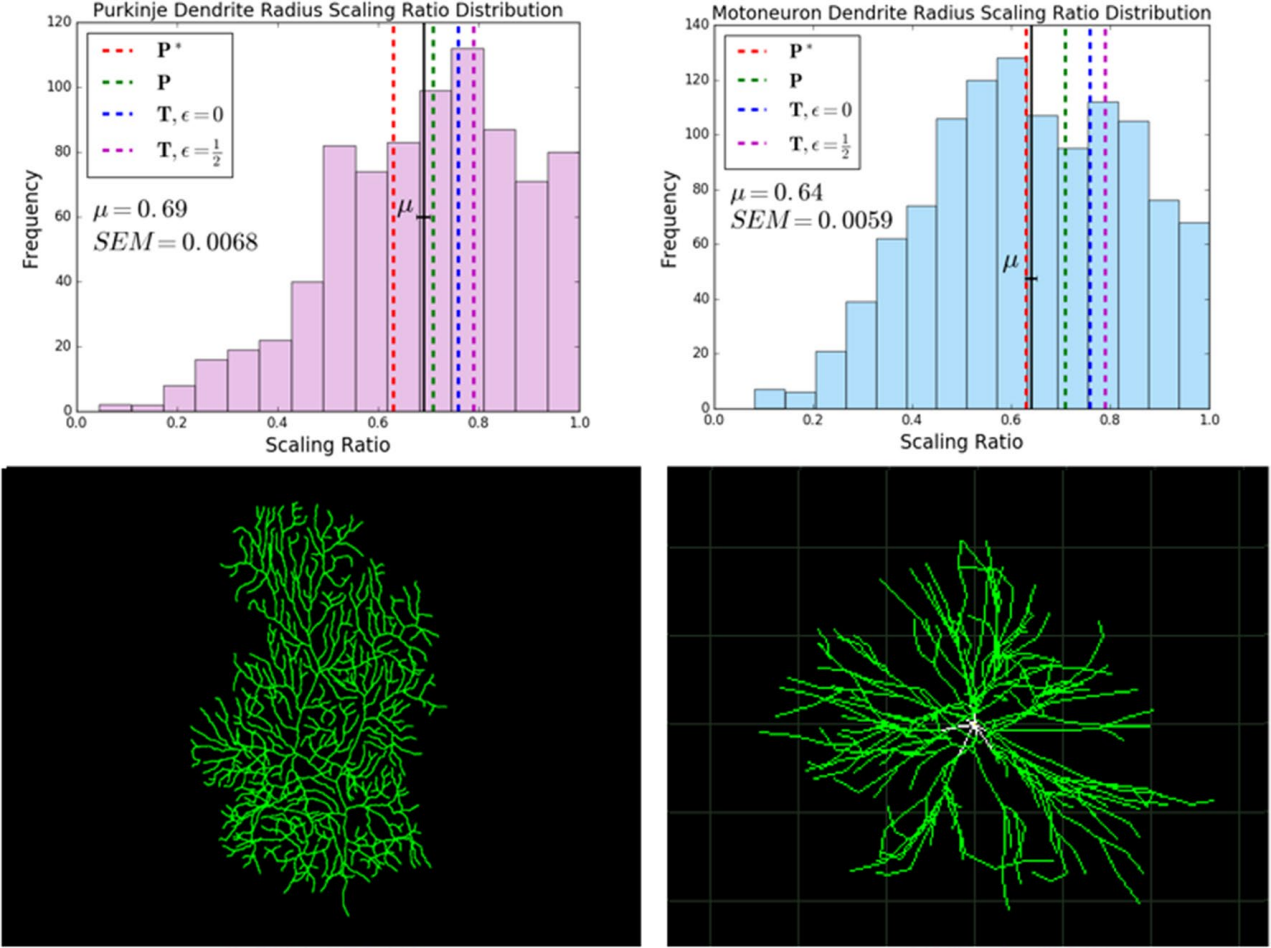
Comparison of radius scaling ratio distributions of cerebellar purkinje cell and motoneuron dendrites. A comparison of histograms showing the distribution of radius scaling ratios observed in dendrites of Purkinje cells and motoneurons, along with representative images. For Purkinje cells, we observe an average radius scaling ratio of $0.69 \pm 0.01$, and for motoneurons, we observe an average radius scaling ratio of $0.64 \pm 0.01$. In the figure, $\mu$ represents the mean and *SEM* represents the standard error of the mean. The standard deviations of the distributions are 0.19 for Purkinje Cells and 0.20 for motoneurons. We have restricted radius scaling ratio data to values that are less than 1.0. The black solid lines denote the mean values in the distributions, shown with error bars, and the red, green, blue, and magenta dashed lines represent the theoretical predictions for various objective functions. The closest theoretical prediction for Purkinje cells is the optimal scaling ratio for function $\textit{P}$, minimizing power with fixed volume, $n^{-1/2} \approx 0.71$. The closest theoretical prediction for motoneurons is the optimal scaling ratio for function $\textit{P*}$, minimizing power with fixed time delay, $n^{-2/3} \approx 0.63$. The representative image for the Purkinje cell is from a mouse^33^ and the representative image for the motoneuron is from a cat spinal motoneuron^39^.

**Figure 4:**
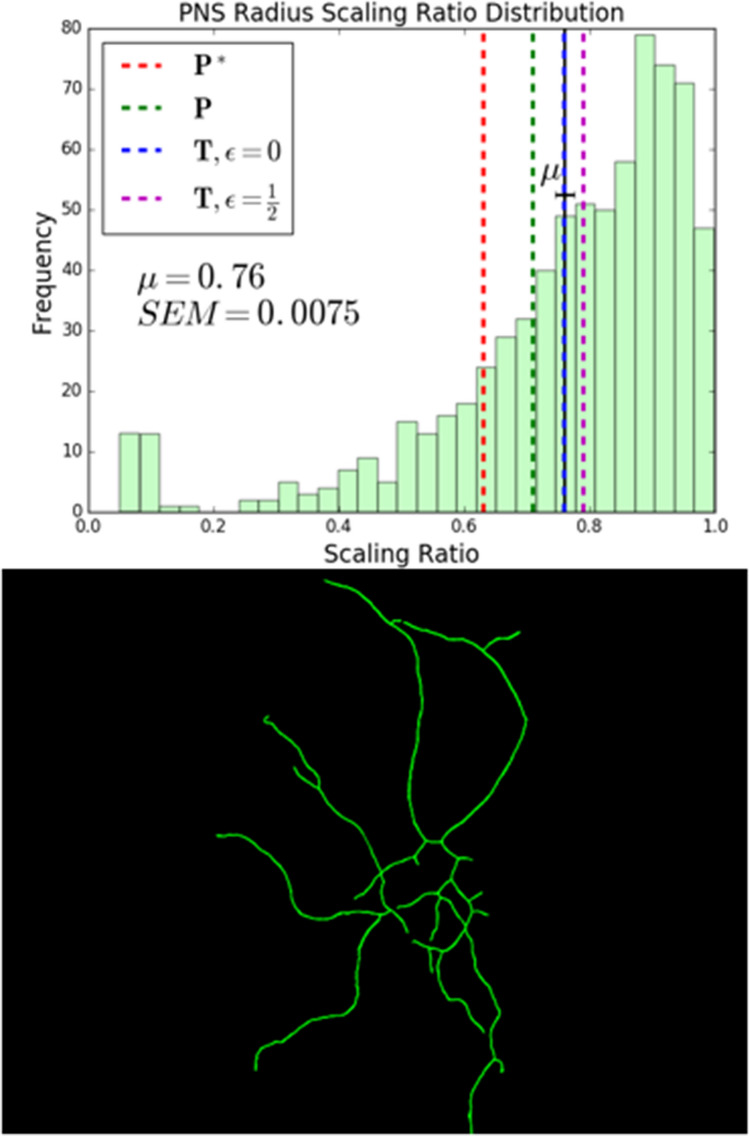
Peripheral nervous system neurons. A histogram showing the distribution of radius scaling ratios in Peripheral Nervous System (PNS) neurons, along with a representative image of the dendritic tree of a mouse sensory neuron^55^. We observe an average radius scaling ratio of $0.76 \pm 0.01$. In the figure, $\mu$ represents the mean and *SEM* represents the standard error of the mean. The standard deviation of the distribution is 0.20. We have restricted radius scaling ratio data to values that are less than 1.0. The black solid lines denote the mean in the distributions, shown with error bars, and the red, green, blue, and magenta dashed lines represent the theoretical predictions for various objective functions. The closest theoretical prediction is $n^{-2/5} \approx 0.76$, the optimal scaling ratio for the function $\textit{T}$ that minimizes time delay for unmyelinated fibers, $\epsilon = 0$.

To test the theoretical predictions and model, it is important to look at empirical data for scaling ratios for radius between child and parent branches in successive levels. We analyzed data from NeuroMorpho.Org—an online database with digital reconstructions from a wide range of species^25^. In this dataset, we have included reconstructions from neuron images using light microscopy methods as well as electron microscopy (EM) images made available through researchers involved with recent projects such as the FlyEM project at Janelia ^26,27^. Figures 2, 3 and 4 show five examples of images of neuron reconstructions obtained from NeuroMorpho.Org. These reconstructions are obtained by tracing neuron image stacks obtained using various microscopic and staining techniques for in vitro neurons and slicing at regular intervals. This database provides 3D reconstruction data that are organized in text files by pixels, in files that specify a pixel ID label for each point, the x, y, z spatial coordinates, the radius of the fiber at each point, and a parent pixel ID, referring to the adjacent pixel previously labelled. The scaling ratios for radius and length can be obtained by organizing this data in terms of branches. This is accomplished by finding the pixels at which the difference between the child pixel ID and the parent pixel ID is greater than 2, which can be defined as branching points. Based on the branching points, a branch ID and parent branch ID can be assigned to each of the pixels.

The radius can be extracted from each of the branches by taking each of the radius values in each branch and averaging them by the following formula, defining each branch as branch k, where the pixels i range from 1 to $N_k$, and where $N_k$ is the last pixel of each branch

14 $$\begin{aligned} r_k = \sum _{i=1}^{N_k} \frac{r_i}{N_k} \end{aligned}$$

The length of each branch can be extracted by summing up the Euclidean distances between each of the points in the branch by the following formula:

15 $$\begin{aligned} l_k = \sum _{i=1}^{N_k} \sqrt{(x_{i}-x_{i-1})^2 + (y_{i}-y_{i-1})^2 + (z_{i}-z_{i-1})^2} \end{aligned}$$

Once the radius and length of each of the branches is found, the scaling ratios are computed by dividing the daughter radius by the corresponding value for the parent branch. Through this method and using the Python library matplotlib, we generate histograms to visualize the distributions. For the radius distributions, we find a large peak at $\frac{r_{k+1}}{r_k} = 1.0$, which is likely due to the resolution limit of the images. After a certain level, the radius for each of the branches is equivalent to the pixel size itself. Thus, in our distributions for radius, we focused on the data for scaling ratios that are less than 1.0. We use solid black lines to denote the mean values in the data, and error bars represent twice the Standard Error of the Mean (SEM)—the standard deviation divided by the square root of the number of data points.

We looked at neuron reconstructions from both axons and dendrites, and from a range of cell types, brain regions, and species. More detailed information about the source of each of the individual reconstructions can be found in Supplementary Information: Text  S4.

For dendrites, we looked at three different types of cells: Golgi cells, Purkinje cells, and motoneurons. The Golgi cells are from *Giraffa*, *Homo Sapiens*, *Loxodonta africana*, *Megaptera novaeangliae*, *Neofelis nebulosa*, *Pan troglodytes*, *Panthera tigris*^28^, and *Mus musculus*^29^. The Purkinje cells are from *Cavia porcellus*^30^, *Mus musculus*^31–34^, and *Rattus*^34–36^. The motoneurons are from *Danio rerio*^37,38^, *Drosophila melanogaster*^26^, *Felis Catus*^39^, *Mus musculus*^40^, *Oryctolagus cuniculus*^41^, *Rattus*^42^, and *Testudines*^43^. In Fig. 2, we look at the combined dendrite data for all cell types and species. In Fig. 3, we look at the radius scaling ratios of Purkinje cells and motoneurons individually, and draw comparisons between the two.

Due to the small size of axons and the limited resolution of images, the data available on NeuroMorpho.Org are limited in scope. The data shown in Fig. 2 was taken from the following species: *Anisoptera*^44^, *Brachyura*^45^, *Drosophila melanogaster*^46^, *Gallus gallus domesticus*^47^, and *Rattus*^48^. The neurons were taken from a range of brain regions: the midbrain, the hippocampus, the antennal lobe, the optic lobe, and the ventral nerve cord.

To study peripheral nervous system neurons, we sampled from reconstruction data that was labelled by region on NeuroMorpho.Org. This data, shown in Fig. 4, was taken from *Drosophila melanogaster*^49–51^ and *Mus musculus*^52–55^ and includes dendritic arborizations, sensory neurons, somatic neurons, and touch receptors.

**Figure 5:**
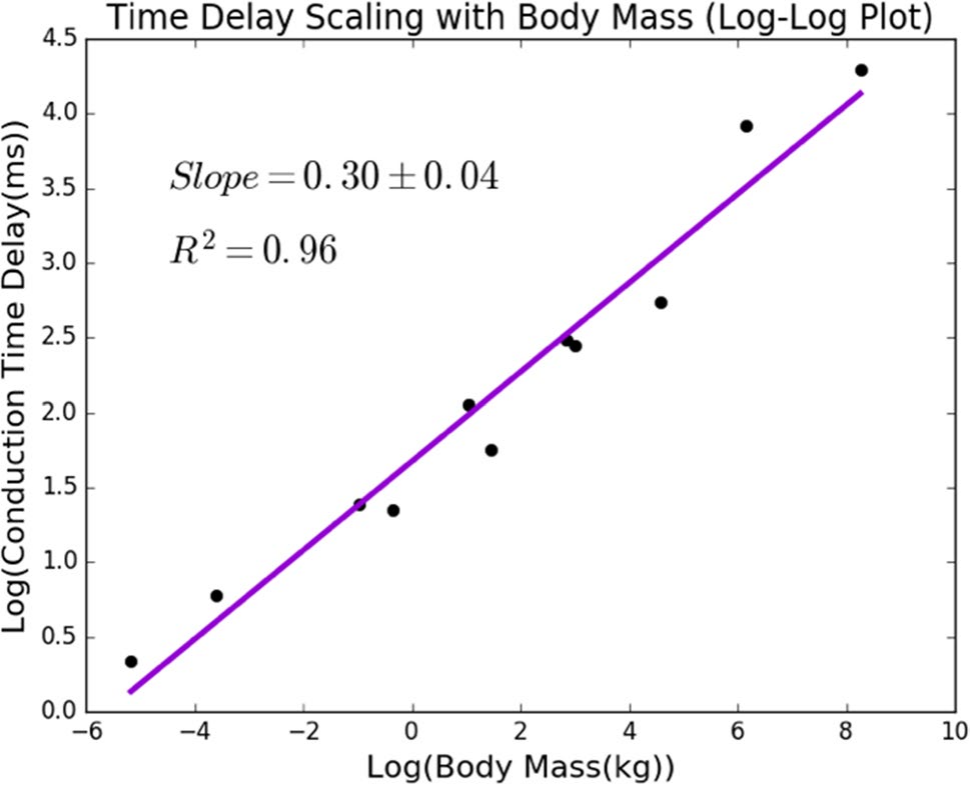
Scaling of conduction time delay and species mass. A scatter plot showing the relationship between the log of the conduction time delay and the log of the body mass of a range of species. Here, the slope, $0.30 \pm 0.04$, corresponds to the power that relates species mass to conduction time delay. This is close to our theoretical result of $\frac{1}{4}$ (= 0.25).

To look at functional scaling relationships between mass and conduction time delay, we first look at data for conduction time delay in motoneurons and sensory neurons across a range of species sizes, listed in order of size: *Soricidae*, *Mus musculus*, *Rattus*, *Cavia porcellus*, *Oryctolagus cuniculus*, *Felis Catus*, *Canis lupus familiaris*, *Sus scrofa*, *Ovis aries*, *Giraffa*, and *Loxodonta africana*^56^. Using the mean conduction velocity measured in studies of each species, this conduction time delay data was calculated by estimating the animal leg length using the average body mass, and then dividing that distance by the measured velocities that vary across species. We use a log-log plot, shown in Fig. 5, to obtain a power law relationship between body mass and conduction time, where the slope is equal to the power.

## Results

We compared theoretical predictions for scaling ratios calculated from objective functions $\textit{T}$, $\textit{P}$, and $\textit{P*}$ with the mean values we measure from the empirical data. Because mean values capture the average overall branching properties for axons and dendrites, the mean represents the most natural and straightforward starting point for comparing our general theory with empirical data. To further clarify our approach in the context of previous studies, most neuroscience studies focus on the $\textit{median values}$ of $\textit{scaling exponent}$ data that are $\textit{exponentially distributed}$, whereas here, we focus on $\textit{scaling ratio}$ data with $\textit{normal}$ and $\textit{symmetric distributions}$, thus rendering the difference between the mean and median values to be negligible. As theory is refined and additional predictions are made, other features of the distribution, such as those related to the spread, should also be measured and compared (see “Discussion”). Based on the results of these comparisons for different types of neurons and processes, we determine the functional properties that play the greatest role in determining structure for different processes and cell types.

### Theoretical predictions

Using the model and the method of undetermined Lagrange multipliers as detailed above, we made theoretical predictions for functions using different values of the parameters. Table 1 shows the results for the various objective functions minimizing conduction time delay and power. The approximations listed are based on the simplifying assumption that the network is purely bifurcating, with a branching ratio of 2.

We consider the theoretical predictions for four objective functions. The first two objective functions are specific cases of $\textit{T}$ (Eq. 2) that minimize conduction time delay. We consider this function for two possible values of the parameter $\epsilon$. The unmyelinated case corresponds to $\epsilon = 0$, whereas $\epsilon = \frac{1}{2}$ signifies the myelinated case. The second two objective functions minimize power. The objective function $\textit{P}$ (Eq. 3) minimizes power with the volume fixed as a constraint. The alternative objective function $\textit{P*}$ (Eq. 4) is the objective function that minimizes power with the time delay fixed as a constraint. For power minimization we focused on the unmyelinated case where $\epsilon$ = 0, since its predictions align most with the data from dendrites, which are typically unmyelinated. These predictions can be transformed into results comparable with existing literature using Eq. (9).

**Table 1:** Results for radius scaling ratio theoretical predictions.

Biophysical principle	Prediction	Closest biological match	Data mean
Time minimization, unmyelinated ($\mathbf {T, \epsilon = 0}$)	$n^{-2/5} \approx 0.76$	Peripheral nervous system neurons	$0.76 \pm 0.008$
Time minimization, myelinated ($\mathbf {T, \epsilon = \frac{1}{2}}$)	$n^{-1/3} \approx 0.79$	Axons	$0.79 \pm 0.001$
Power minimization with fixed volume ($\textbf{P}$)	$n^{-1/2} \approx 0.71$	Purkinje cell dendrites	$0.69 \pm 0.007$
Power minimization with fixed time delay ($\mathbf {P^*}$)	$n^{-2/3} \approx 0.63$	Motoneuron dendrites	$0.64 \pm 0.006$

For all of the calculations, we considered different values of the parameter *d*, the dimension of space filled by the processes. A value of *d* = 2 signifies neuron processes that branch into a 2-dimensional plane, such as Purkinje cells in the cerebellum. A value of *d* = 3 signifies neuron processes that fill a 3-dimensional volume, such as motoneurons^6^. We interpreted the volume constraint as a material constraint, assuming that the processes are cylindrical for both 2- and 3-dimensional space-filling. It is interesting to note that the dimension of space filling does not affect results for radius scaling ratios. However, it does play a role in the results of the theoretical predictions for length. As in the studies of cardiovascular network branching, we focused on radius scaling ratios in this analysis^57^. We explain this choice further in “Discussion”.

### Dendrites and axons

Figure 2 shows histograms that illustrate the differences in distributions of radius scaling ratios for dendrites and axons, along with representative images of the morphology of these two processes. Axons generally carry signals from the cell body to the synapses, where they transfer information to the dendrites of other neurons. Dendrites have extensive, tree-like structures and generally connect with the axons of other neurons to carry signals to the cell body. The distributions observed for these scaling ratios resemble the distributions observed in scaling ratios of cardiovascular networks, with the radius scaling ratios exhibiting a normal distribution. Note that the distribution of scaling ratios for axons is normal, but we have restricted it to values between 0 and 1, eliminating the cases where the resolution limit of the images is reached and the values greater than 1, which are biologically questionable.

In this figure, we show the comparison of the mean dendrite radius scaling ratio, $0.67 \pm 0.004$, with theoretical predictions from the four different calculations. We find that the dendrite radius scaling ratio mean is closest to the theoretical predictions from the objective functions minimizing power. The mean lies in between the optimal scaling ratios for function $\textit{P}$, so $n^{-1/2} \approx 0.71$, which holds volume to be fixed, and function $\textit{P*}$, so $n^{-2/3} \approx 0.63$, which holds time delay to be fixed. Later, in Fig. 3 we look at the distributions of radius scaling ratios in these Purkinje cells and motoneurons individually to compare them to the closest theoretical results. In “Discussion”, we elaborate on the implications of focusing on the means in this analysis over the spread of the distributions.

Note that the radius scaling ratio mean for axons, $0.79 \pm 0.001$, is significantly larger than the mean radius scaling ratio observed for dendrites, $0.67 \pm 0.004$. The axon scaling ratio mean in the data is closest to the theoretical prediction, $n^{-1/3} \approx 0.79$, for the objective function that minimizes time, $\textit{T}$, for myelinated fibers, $\epsilon = \frac{1}{2}$. The next closest prediction, $n^{-2/5} \approx 0.76$, is that of the objective function that minimizes time, $\textit{T}$ for unmyelinated fibers, $\epsilon = 0$. This suggests that time minimization and myelination are important factors that determine the structure for axons.

### Purkinje cells and motoneurons

One of the parameters that we built into our theoretical model is *d*, the dimension of space filling of the processes. Thus, we looked at the comparison of results from data from representative cells with 2-dimensional and 3-dimensional dendritic trees. For 2-dimensional dendritic trees, we looked at cerebellar Purkinje cell data from rodents including mice, rats, and guinea pigs. For 3-dimensional dendritic trees, we looked at motoneurons from a range of species including rodents, amphibians, cats, and humans. The histograms for these two cell types, along with a representative image for each type, are shown in Fig. 3. The theoretical results of minimizing power and time cost functions while varying the parameter *d* does not capture the differences in radius scaling ratios observed in this data. We hypothesized that the differences observed can be explained by other principles such as the functional differences of these cell types. The mean for Purkinje cells, $0.69 \pm 0.01$, agrees with the theoretical predictions for the function $\textit{P}$, that is power minimization with a volume constraint, $n^{-1/2} \approx 0.71$, while the mean for motoneurons, $0.64 \pm 0.01$, agrees with the theoretical predictions for function $\textit{P*}$, that is power minimization with a time constraint, $n^{-2/3} \approx 0.63$.

Based on the results of the comparison of Purkinje cells and motoneurons, we concluded that volume plays a greater role in constraining the structural design of Purkinje cells, while time plays a greater role in constraining the structural design of motoneurons.

### Peripheral nervous system neurons

In the peripheral nervous system (PNS), motoneurons play an important role in the exchange of information with sensory neurons. Peripheral nerves carry sensory information and interact with motoneurons, which directly innervate effector cells such as muscles^6^. Thus, the importance of conduction time as a constraint for motoneurons motivated us to examine data from other types of PNS neurons such as sensory neurons. Figure 4 shows the radius scaling distribution of a sample of the PNS neurons labelled by region on NeuroMorpho.Org. This data was taken from flies and mice. The mean radius scaling ratio, $0.76 \pm 0.01$, is closest to the theoretical prediction, $n^{-2/5} \approx 0.76$, for the objective function, $\textit{T}$, that minimizes time for unmyelinated fibers, $\epsilon = 0$. This suggests that time is an important factor in optimizing structure for PNS neurons.

### Time delay scaling

So far, we have focused on predictions and data combined from species of a range of sizes. Here, we consider how function varies across species of a range of body masses. We used $\textit{P*}$, the equation minimizing power with fixed time delay. As shown in “Allometry calculation”, our theoretical calculations have led to the relationship between conduction time delay and mass described in Eq. (13).

In order to test this theoretical result, we analyzed experimental data to determine an observed relationship between time delay as a function of species size. Previous experimental studies have looked at conduction time delay across species ranging from shrews to elephants^56^. A regression analysis of the data shows that the $\frac{1}{4}$-power mass term is more significant than the linear term, as is shown in more detail in Supplementary Information: Text  S3. Furthermore, we used a log-log plot to determine the power of the relationship, plotting the log of the conduction time delay data against the log of the average body mass of each species. This plot is shown in Fig. 5.

Our theoretical predictions suggest the presence of a $\frac{1}{4}$ (= 0.25) power law that relates species mass to neuron conduction time delay. These experimental results support this power law, as the power law determined from the data is $0.30 \pm 0.04$. It is possible that at a wider range of masses, a scaling law closer to the linear relationship might be observed. Further data and analysis of the relationship between the size of individual neurons and processes and species mass and between conduction velocity and time delay will provide useful insight into this allometry.

## Discussion

A comparative analysis of the radius scaling ratios of different processes and cell types suggests that there are selection pressures for different functional roles that underlie the diversity in neuron branching patterns. There are a number of characteristic differences observed between axons and dendrites that are maintained across species and cell size. Axons are long and function to transmit signals over large distances, sometimes between different regions of the nervous system^58^. Moreover, axons have the unique property of myelination, which provides an important role in information transfer in the nervous system^59^. Our results indicate that the radius scaling ratio mean for axons is closest to the prediction that minimizes time for conduction through myelinated fibers, which supports this notion that information processing speed is a key principle governing the structure of axons. The predictions and results from the data for axons are supported by previous theoretical results predicting scaling laws for myelinated axons^21^.

In contrast, dendritic trees are relatively short, have more extensive branching, and generally do not conduct action potentials^58^. Previous theoretical work on wiring optimization in cortical circuits similarly proposes that there are differing evolutionary selection pressures governing axons and dendrites. Rather than conduction time delay, the key principle behind dendritic structure is passive cable attenuation^60^. Our results suggest that dendrites are optimized to minimize power, which is related to a voltage drop, with a volume constraint that we have interpreted as a cost in materials. Thus, minimizing power in our theoretical framework is effectively minimizing the attenuation of the passive signals in dendrites.

There is a great deal of diversity in the branching structures of dendritic trees, and the differences in scaling ratio distributions among the different types gives us important insights into their distinct functional roles. We found that the structure of Purkinje cells and motoneurons are both governed by power minimization, and Purkinje cell structure is constrained by volume while motoneuron structure is constrained by time delay. The predictions and results from the data for Purkinje cells and motoneurons are supported by previous theoretical and experimental results^20,61^. We conclude that time plays a greater role in optimizing the structure for motoneuron dendrites.

Efficiency in information processing is a key function of neurons in the sensorimotor system, and our results emphasize that function as a key feature governing their structural design. When organisms are exposed to environmental stimuli, it triggers a response in the motor system that must be executed very rapidly. Some of these responses are innate, and some are learned through practice, gradually increasing in speed^62^. We found that the structure of neurons in the peripheral nervous system, such as the sensory neurons that relay information from the environment to motoneurons, is governed by time minimization, which is consistent with the evolutionary function of the sensorimotor system. The correspondence of our theoretical predictions with empirical measurements from neurons of different types supports intuitive notions about neuron computation in these specific cell types.

So far, we have looked at optimization problems minimizing power and time individually. However, it is possible that there might be intermediate values, and different cell types might have different relative importance of time and power in determining structure. A possible avenue for future work is using numerical methods to extend the number of functional principles we consider and to better estimate parameters, such as the relative importance of different functional principles and degree of myelination. This might provide a more biologically realistic estimate for scaling ratios, as it is likely that neuron cell structures are designed to optimize not only conduction speed or energy efficiency, but a relative combination of both.

Recent work has looked at data and the scaling exponents in dendrites to compare the results to Rall’s law for neurons—with a 3/2 exponent to describe branching diameters—as well as exponents derived for other biological networks such as cardiovascular networks and trees. Their data show a range of exponents varying from Rall’s law, and they propose that cell biological constraints related to intracellular transport and the cytoskeleton are important in determining the morphology of neurons^63^. This is supported by other recent studies that relate dendritic morphology, including measures of caliber, to cytoskeletal proteins such as microtubules and actin filaments^64,65^. While it is likely that these are important considerations driving morphology, our general model derives scaling ratios with a range of values—depending on the tradeoff of functional principles—that agree with the median range of exponents in their data, suggesting that our framework is a promising general model that accounts for much of the observed variation around Rall’s law.

The similarities in distributions of scaling ratios in radius and length between neurons and cardiovascular networks suggest that a unifying framework underlies these diverse biological systems. Moreover, our work extends previous work on biological scaling theory in resource distribution networks by considering other driving factors besides energy efficiency as well as the tradeoff between multiple functional principles. This approach and the inclusion of additional principles has the potential to motivate future studies both in neuroscience and biological resource distribution networks.

Some features of neural systems not captured by our current model could be incorporated in future iterations. For instance, the morphology of dendritic arbors is not static but is constantly changing based on interactions with surrounding neurons and glia^6^. Moreover, we have formulated the space-filling constraint based on the idea that cardiovascular networks are optimized such that vessels feed every cell in the body. However, neurons exhibit more complex space-filling patterns due to their interactions with one another, such as tiling and self-avoidance^66,67^. It might also be fruitful to consider different formulations of the space-filling constraints for different types of neurons. For example, axons tend to have projections that feature a longer parent branch, and the daughter branches occur further away from the soma. Indeed, previous work has extended the WBE model to look at scaling in plants^68^. Previous work on space-filling for plants such as palm trees, which have similar morphology, might help guide future studies in this direction and improve predictions, particularly for length scaling ratios.

We have have chosen to focus on radius scaling ratios in this analysis because the branch length measurements are not accurately characterized, as also previously reported for vascular scaling^57^. Recent work suggests Horton-Strahler labeling—where the first level begins at the tips, and higher levels are determined when two branches of the same level combine—may yield better estimates of branch length scaling^69^. For instance, previous work on river networks has used Horton–Strahler labeling, and it has been applied to other networks in biology, particularly those in in which asymmetric branching is observed^70,71^.

Hermann Cuntz’s group has also applied this ordering method to analyze dendritic trees, finding differences in branching metrics across neuron cell types^72^. In future work, we plan to investigate how this alternative labeling scheme for branch lengths compares with theoretical predictions derived using our framework. We hypothesize that applying this labeling scheme to define branching levels for length will give a distribution of scaling ratios that looks more like the normal distributions observed for radius scaling ratios, and values for means that agree more closely with our theoretical predictions, as has been seen in work on cardiovascular networks.

Furthermore, we note that our comparison of the predictions to the data involve only the mean values. The mean provides a single, simple, cumulative, and easily interpretable measure. Other possible choices include the mode of the distributions, which would differently account for the spread or the shape of the variation of the distributions. The mean and mode values do not align in all cases. Future work should look further into additional features—such as variance or higher-order moments—of the distributions of radius and length scaling ratios in order to extract even more valuable information from the data.

Additionally, we have represented the energy consumption here as the power lost due to dissipation during signaling. In neurons, however, maintaining the resting membrane potential makes up a significant fraction of the energetic costs. Here, we assumed that this cost is captured in the volume term in the model. However, it might be possible to more explicitly formalize the inclusion of the resting potential via the incorporation of additional factors that affect this cost. For example, myelination affects the surface area as well as the capacitance of axons, and the energy required to maintain the resting potential varies linearly with capacitance^18^. Incorporating these complexities in our model might improve its biological accuracy and usefulness when comparing predictions to empirical data from neurons.

Throughout this model, we have assumed that branching is symmetric—the radius and length of daughter branches are identical. Previous work has attempted to capture asymmetry in cardiovascular networks and plants^73^. Another major goal of our future work is to apply this theoretical framework to look at branching of neuron processes and to use branching properties related to asymmetry to compare different cell types.

Beyond the scaling ratios for successive branches in the individual neuron processes, it is interesting to consider allometric scaling relationships of species size and functional properties that vary with size. Previous work on cardiovascular networks has extracted an allometric scaling relationship that relates species size (or mass) with network volume^9^, and other previous work on scaling has shown an allometric scaling relationship between single cell neurons and animal body mass^10^. In addition, when brains grow in size, they require more extensive axonal trees to traverse greater distances^74^. Building on these ideas from our theoretical formulation of the objective function that minimizes power subject to the constraint of fixed conduction time delay, we were able to extract a functional scaling relationship between species size and time delay for unmyelinated fibers. We derived that there is a mixed power law relationship between animal body mass and conduction time delay, including both a term with a $\frac{1}{4}$-power and a linear term with mass. The dominance of the $\frac{1}{4}$-power law is supported by experimental data of conduction time delay from species of a range of masses: the conduction time delay scales with the fourth root of the animal body mass.

An interesting aspect of this result is that neurons in larger animals have longer conduction delays. These results are important to consider in the context of evolution—longer delays might provide a functional explanation for the increased specialization of brain function hemispheres. Due to the greater conduction time delays, it might be advantageous for larger brains to exhibit more specialization and to organize cells with information about related memories and skills in localized clusters^24^, thus improving the efficiency of information processing.

## Conclusion

We conclude that neuron function places profound constraints on neuron morphology, thus cementing the foundations in Ramón y Cajal’s work^3^ and resulting theoretical and computational formalism by Cuntz and Chklovskii^12,60^. We extend this work to include metabolic constraints and to consider the volumetric aspect of morphology. Our approach provides a framework to measure and quantify neuron morphology, and a mathematically and theoretically advanced way to describe the influence of biophysical constraints in selecting morphological patterns in neurons. Combining empirical measures with our theoretical predictions, we showed fundamental differences between axons and dendrites and between Purkinje cells and motoneurons that are connected to the myelination of axons and the dimension of space being filled by the branching processes. Our results are consistent with and supportive of the hypothesis that the tradeoff between these functional principles governs neuronal branching and structure, and therefore accounts for the variation in scaling laws observed in recent studies^63^. Future work will shed even more light on these foundational questions by building models to capture more biological complexity and by obtaining larger amounts of data at higher resolutions across more species and more cell types. Indeed, looking across species and cell types will also help reveal further differences in neuronal function and tradeoffs among different principles that may transform how we understand the function and form of the brain.

## Supplementary Information

Supplementary Information.

## Data Availability

Data are available at www.neuromorpho.org. Supplementary Information: Text S4 details the file names of the cells we analyzed, including the cell type, region, species, and archive from which the data were taken.
