# Supplementary material for: How axon and dendrite branching are guided by time, energy, and spatial constraints
Source: Sci Rep. 2022 Dec 2;12:20810. doi: 10.1038/s41598-022-24813-2 (PMC9718790; doi:10.1038/s41598-022-24813-2)
Supplement: Supplementary file 1 — Supplementary Information. [file 41598_2022_24813_MOESM1_ESM.pdf]

Supplementary Information

How Axon and Dendrite Branching Are Governed by Time, Energy, and Spatial Constraints

Paheli Desai-Chowdhry[1]*, Alexander B. Brummer[1,2] ¤, Van M. Savage[1,2,3]

1 Department of Computational Medicine, University of California Los Angeles, Los Angeles, California, United States of America
2 Department of Ecology and Evolutionary Biology, University of California Los Angeles, Los Angeles, California, United States of America
3 Santa Fe Institute, Santa Fe, New Mexico, United States of America

Text S1. Scaling Ratio Calculation

We use the method of Lagrange multipliers to solve for the values of the scaling ratios for radius and length, $\frac{r_{k+1}}{r_k}$ and $\frac{l_{k+1}}{l_k}$, that minimize the objective function. This is carried out by setting the derivatives of these functions - with respect to radius and length - equal to 0 and solving for the Lagrange multipliers. These values are assumed to be constant, so we solve for the scaling ratios by setting the ratio of the multiplier expressions at successive branching generations equal to 1.

Below, we show a sample calculation of the method of Lagrange multipliers for one of the cases. We will consider the objective function P - the function minimizing power with fixed volume, mass, and space filling - for our sample calculation. Below is the equation for this function:

$$P = \sum_{k=0}^{N} \frac{l_k}{r_k^2 n^k} + \lambda \sum_{k=0}^{N} n^k r_k^2 l_k + \lambda_m m_c + \sum_{k=0}^{N} \lambda_k n^k l_k^d \tag{S1.1}$$

Radius Scaling Ratio Calculation

To find the radius scaling ratio, we will minimize P with respect to r_k, at an arbitrary level k, and set the result equal to 0. Thus, we can find a formula for a Lagrange multiplier and derive the scaling law.

$$\frac{\partial P}{\partial r_k} = \frac{-2l_k}{n^k r_k^3} + 2\lambda n^k r_k l_k = 0 \tag{S1.2}$$

Solving for the Lagrange multiplier, we have

$$\lambda = \frac{1}{n^{2k} r_k^4} \tag{S1.3}$$

Since this is a constant, the denominator must be constant across levels.

$$\frac{n^{2(k+1)} r_{k+1}^4}{n^{2k} r_k^4} = 1 \tag{S1.4}$$

Thus, we can solve for the scaling ratio

$$\frac{r_{k+1}}{r_k} = \left(n^{-2}\right)^{1/4} = n^{-1/2} \tag{S1.5}$$

Length Scaling Ratio Calculation

To find the length scaling ratio, we will minimize P with respect to l_k, at an arbitrary level k, and set the result equal to 0. Thus, we can find a formula for a Lagrange multiplier, using the formula above, and derive the scaling law.

$$\frac{\partial P}{\partial l_k} = \frac{1}{n^k r_k^2} + \lambda n^k r_k^2 + d\lambda_k n^k l_k^{d-1} = 0 \tag{S1.6}$$

Solving for the Lagrange multiplier, we have

$$\lambda_k = \frac{-\frac{1}{n^k r_k^2} - \lambda n^k r_k^2}{d n^k l_k^{d-1}} \tag{S1.7}$$

Substituting λ, as calculated before, we can simplify the expression for this multiplier as follows

$$\lambda_k = \frac{-\frac{1}{n^k r_k^2} - \frac{1}{n^k r_k^2}}{d n^k l_k^{d-1}} = -\frac{2}{d n^{2k} l_k^{d-1} r_k^2} \tag{S1.8}$$

Since this is a constant, the denominator must be constant across levels, so

$$\frac{n^{2(k+1)} l_{k+1}^{d-1} r_{k+1}^2}{n^{2k} l_k^{d-1} r_k^2} = 1 \tag{S1.9}$$

Thus, substituting in the scaling ratio for radius, we can solve for the scaling ratio for length

$$\left(\frac{l_{k+1}}{l_k}\right)^{d-1} = n^{-2} \left(\frac{r_{k+1}}{r_k}\right)^{-2} \tag{S1.10}$$

$$\left(\frac{l_{k+1}}{l_k}\right)^{d-1} = n^{-2} \left(n^{-1/2}\right)^{-2} = n^{-1} \tag{S1.11}$$

For the case where the dimension of space filling, d, is equal to 3, we have

$$\frac{l_{k+1}}{l_k} = n^{-1/2} \tag{S1.12}$$

This method is repeated to solve for the theoretical predictions of scaling ratios for radius and length for the other objective functions.

Text S2. Allometry Calculation

We can use the objective function P^* - the function minimizing power with fixed time delay, size, and space filling - to derive a functional scaling relationship between conduction time delay and species mass, considering the unmyelinated case where ϵ is equal to 0, and the case of 3-dimensional space filling, choosing d to be 3. The equation for this function is

$$P^* = \sum_{k=0}^{N} \frac{l_k}{r_k^2 n^k} + \lambda \sum_{k=0}^{N} \frac{l_k}{r_k^{\frac{1}{2}}} + \lambda_m m_c + \sum_{k=0}^{N} \lambda_k n^k l_k^3 \tag{S2.1}$$

We begin by setting the derivative of the function with respect to radius equal to zero to solve for the multiplier λ.

$$\frac{\partial P^*}{\partial r_k} = \frac{-2l_k}{r_k^3 n^k} - \frac{\lambda l_k r_k^{-3/2}}{2} = 0 \tag{S2.2}$$

Below, we have the expression for the multiplier

$$\lambda = \frac{-4}{r_k^{3/2} n^k} \tag{S2.3}$$

We can similarly solve for the multiplier λ_k by setting the derivative with respect to length equal to 0.

$$\frac{\partial P^*}{\partial l_k} = \frac{1}{r_k^2 n^k} + \lambda r_k^{-1/2} + 3\lambda_k n^k l_k^2 = 0 \tag{S2.4}$$

Using the expression for λ above, we can solve for an expression for λ_k.

$$\lambda_k = \frac{1}{r_k^2 n^{2k} l_k^2} \tag{S2.5}$$

If we plug this expression for λ_k back into the original expression for P^*, we get

$$P^* = \sum_{k=0}^{N} \frac{l_k}{r_k^2 n^k} + \lambda \sum_{k=0}^{N} \frac{l_k}{r_k^{1/2}} + \lambda_m m_c + \sum_{k=0}^{N} \left(\frac{1}{r_k^2 n^{2k} l_k^2} \right) n^k l_k^3 \tag{S2.6}$$

The last term simplifies to a term that is identical in form to the power term. So we can rewrite this as

$$P^* = 2 \sum_{k=0}^{N} \frac{l_k}{r_k^2 n^k} + \lambda \sum_{k=0}^{N} \frac{l_k}{r_k^{1/2}} + \lambda_m m_c \tag{S2.7}$$

For simplicity, if we denote the power expression as P, the time delay expression as T, we can rewrite this as

$$P^* = 2P + \lambda T + \lambda_m m_c \tag{S2.8}$$

Previous results have shown a proportional relationship between m_c, the mass of a single neuron, and the fourth root of an animal's body mass, $M^{1/4}$ [Savage et al, 2007]. Thus, we can replace this term and consider a new Lagrange multiplier with the absorbed constant

$$P^* = 2P + \lambda T + \lambda_M M^{1/4} \tag{S2.9}$$

We will now take the derivative of this term with respect to M, the mass of the species, and set it equal 0.

$$\frac{\partial P^*}{\partial M} = 2\frac{\partial P}{\partial M} + \lambda\frac{\partial T}{\partial M} + \lambda_M \frac{\partial M^{1/4}}{\partial M} = 0 \tag{S2.10}$$

Previous results have shown that the energetic cost, which we have interpreted here as power loss due to dissipation, decreases with increasing body weight of animals at a linear rate [Wang et al., 2008]. Thus, we can express $\frac{\partial P}{\partial M}$ generally as a negative constant, $-C$. We can rewrite the above expression as

$$\frac{\partial T}{\partial M} = \frac{-\lambda_M M^{-3/4}}{4\lambda} + 2\frac{C}{\lambda} \tag{S2.11}$$

Solving this differential equation, we have

$$T = \frac{-\lambda_M}{\lambda} M^{1/4} + \frac{2C}{\lambda} M + C_0 \tag{S2.12}$$

If we apply the initial condition T=0 for M =0, we get $C_0 = 0$. Thus, we obtain the following expression relating conduction time delay and body mass

$$T = \frac{-\lambda_M}{\lambda} M^{1/4} + \frac{2C}{\lambda} M \tag{S2.13}$$

Text S3. Allometric Scaling Relationship Regression Analysis

Our calculations have led to the following allometric relationship between conduction time delay and species mass

$$T = \frac{-\lambda_M}{\lambda} M^{1/4} + \frac{2C}{\lambda} M \tag{S3.1}$$

Note that the function for conduction time delay is a linear combination of two terms. The first term depends on the $\frac{1}{4}$-power of the body mass and the second term depends linearly on the body mass.

In order to test the fit of this model to the data, we will run a regression analysis on the following linear model

$$T = \beta_0 + \beta_1 M + \beta_2 M^{1/4} \tag{S3.2}$$

Table C.1. Regression Coefficients

	**Estimate**	**Standard Error**	**t value**	**Pr($>|t|$)**
(Intercept)	-4.79	3.20	-1.50	0.172
M	0.00132	0.00326	0.408	0.694
$M^{1/4}$	9.40	1.66	5.66	0.000478

Estimated coefficients for each term in a linear model fitting conduction time delay to M and $M^{1/4}$ shows the relative weight of each of the terms in the model as well as the likelihood that the relationship between the term and conduction time delay is purely by chance. The notation Pr($>|t|$) represents the p-values, or the probability that the correlation observed is due to random variation.

Here, β_0, β_1, and β_2 are the estimated coefficients. This will allow us to estimate the magnitude of each of these coefficients and the relative importance of each term in determining the conduction time, based on data.

Below is a summary of the results

These results suggest that the $M^{1/4}$ term dominates in terms of magnitude, as its coefficient, 9.40, is higher than the coefficient for the linear mass (M) term that is 0.00132. Moreover, the Pr($>|t|$) or p-values suggest that the $M^{1/4}$ term is the only term that is not likely to be due to random chance.

Text S4. Overview of Reconstruction Data Sources

Table 1. NeuroMorpho.Org Reconstruction Data: Golgi cells

Cell Type	Region	Species	Archive Name	File Name
Golgi cells	Cerebellum	*Giraffa*	Jacobs	185-4-4dw
Golgi cells	Cerebellum	*Giraffa*	Jacobs	186-4-7dw
Golgi cells	Cerebellum	*Giraffa*	Jacobs	187-4-1dw
Golgi cells	Cerebellum	*Homo Sapiens*	Jacobs	189-1-21dw
Golgi cells	Cerebellum	*Homo Sapiens*	Jacobs	189-1-25dw
Golgi cells	Cerebellum	*Homo Sapiens*	Jacobs	189-1-29dw
Golgi cells	Cerebellum	*Loxodonta africana*	Jacobs	155-1-2Gol
Golgi cells	Cerebellum	*Loxodonta africana*	Jacobs	155-2-6Gol
Golgi cells	Cerebellum	*Loxodonta africana*	Jacobs	155-4-5Gol
Golgi cells	Cerebellum	*Megaptera novaeangliae*	Jacobs	202-2-18nj
Golgi cells	Cerebellum	*Megaptera novaeangliae*	Jacobs	202-2-21nj
Golgi cells	Cerebellum	*Megaptera novaeangliae*	Jacobs	202-2-44nj
Golgi cells	Cerebellum	*Neofelis nebulosa*	Jacobs	195-4-8nj
Golgi cells	Cerebellum	*Pan troglodytes*	Jacobs	205-2-16nj
Golgi cells	Cerebellum	*Pan troglodytes*	Jacobs	205-2-21nj
Golgi cells	Cerebellum	*Pan troglodytes*	Jacobs	205-2-31nj
Golgi cells	Cerebellum	*Panthera tigris*	Jacobs	194-4-19nj
Golgi cells	Cerebellum	*Panthera tigris*	Jacobs	194-4-22nj
Golgi cells	Cerebellum	*Panthera tigris*	Jacobs	194-4-4nj
Golgi cells	Cerebellum	*Mus musculus*	Vervaeke	210710C0
Golgi cells	Cerebellum	*Mus musculus*	Vervaeke	240710C0
Golgi cells	Cerebellum	*Mus musculus*	Vervaeke	Golgi-cell-051108-C0-cell1

A table detailing the identity and sources of the Golgi cell reconstruction data extracted from the online database NeuroMorpho.Org. The standardized Morphology Files were used and manipulated based on the methods described in the main text in order to extract the radius and length scaling ratio distributions.

Table 2. NeuroMorpho.Org Reconstruction Data: Purkinje cells

Cell Type	Region	Species	Archive Name	File Name
Purkinje cells	Cerebellum	*Cavia porcellus*	Dendritica	v_e_purk1
Purkinje cells	Cerebellum	*Cavia porcellus*	Dendritica	v_e_purk2
Purkinje cells	Cerebellum	*Cavia porcellus*	Dendritica	v_e_purk3
Purkinje cells	Cerebellum	*Mus musculus*	Hess	180524_E4_KO
Purkinje cells	Cerebellum	*Mus musculus*	Dusart	Purkinje-slice-ageP35-1
Purkinje cells	Cerebellum	*Mus musculus*	DeMunter	SDM_Purkinje_WT3
Purkinje cells	Cerebellum	*Mus musculus*	Martone	e1cb4a5
Purkinje cells	Cerebellum	*Rattus*	Buffo	1-2-2_18
Purkinje cells	Cerebellum	*Rattus*	Buffo	1-2-8_6
Purkinje cells	Cerebellum	*Rattus*	Martone	alxP
Purkinje cells	Cerebellum	*Rattus*	Dendritica	p19
Purkinje cells	Cerebellum	*Rattus*	Dendritica	p20

A table detailing the identity and sources of the Purkinje cell reconstruction data extracted from the online database NeuroMorpho.Org. The standardized morphology files were used and manipulated based on the methods described in the main text in order to extract the radius and length scaling ratio distributions.

Table 3. NeuroMorpho.Org Reconstruction Data: Motoneurons

Cell Type	Region	Species	Archive Name	File Name
Motoneurons	Spinal Cord	*Danio rerio*	Morsch	1_180107_mnx1_mVenus_taken160715
Motoneurons	Spinal Cord	*Danio rerio*	Morsch	2_180107_mnx1_mKO2CX_taken160808
Motoneurons	Spinal Cord	*Danio rerio*	Morrice	NeuronStudio_VehicleControl_48hpf1
Motoneurons	Optic Lobe	*Drosophila Melanogaster*	Shinomiya_FlyEM	14135943
Motoneurons	Optic Lobe	*Drosophila Melanogaster*	Shinomiya_FlyEM	C3-B_145
Motoneurons	Optic Lobe	*Drosophila Melanogaster*	Shinomiya_FlyEM	2546551
Motoneurons	Optic Lobe	*Drosophila Melanogaster*	Shinomiya_FlyEM	C3-F_19
Motoneurons	Optic Lobe	*Drosophila Melanogaster*	Shinomiya_FlyEM	2583823
Motoneurons	Optic Lobe	*Drosophila Melanogaster*	Shinomiya_FlyEM	C3_Home_10
Motoneurons	Spinal Cord	*Felis Catus*	Burke	v_e_moto1
Motoneurons	Spinal Cord	*Felis Catus*	Burke	v_e_moto4
Motoneurons	Spinal Cord	*Felis Catus*	Burke	v_e_moto5
Motoneurons	Spinal Cord	*Mus musculus*	Leroy	04-04-MN9
Motoneurons	Spinal Cord	*Mus musculus*	Leroy	06-04-MN4
Motoneurons	Spinal Cord	*Mus musculus*	Leroy	06-09-MN
Motoneurons	Spinal Cord	*Oryctolagus cuniculus*	Quinian	KQa11-12-2015-tracing
Motoneurons	Spinal Cord	*Oryctolagus cuniculus*	Quinian	KQa29-3-2016$_3$60
Motoneurons	Spinal Cord	*Oryctolagus cuniculus*	Quinian	KQa8-4-2016-tracing
Motoneurons	Spinal Cord	*Rattus*	Alvarez	Alvarez-Control-Cell-2
Motoneurons	Spinal Cord	*Rattus*	Alvarez	Alvarez-Control-Cell-3
Motoneurons	Spinal Cord	*Rattus*	Alvarez	Alvarez-Regen-Cell-4
Motoneurons	Spinal Cord	*Testudines*	Chmykhova	2T-CMOT
Motoneurons	Spinal Cord	*Testudines*	Chmykhova	5Tmn1
Motoneurons	Spinal Cord	*Testudines*	Chmykhova	5Tmn2

A table detailing the identity and sources of the motoneuron reconstruction data extracted from the online database NeuroMorpho.Org. The standardized morphology files were used and manipulated based on the methods described in the main text in order to extract the radius and length scaling ratio distributions.

Table 4. NeuroMorpho.Org Reconstruction Data: Axons

Cell Type	Region	Species	Archive Name	File Name
Target-Selective Descending	Ventral Nerve Cord	*Anisoptera*	Peng	C150
Target-Selective Descending	Ventral Nerve Cord	*Anisoptera*	Peng	C168
Target-Selective Descending	Ventral Nerve Cord	*Anisoptera*	Peng	C201
Columnar	Optic Lobe	*Brachyura*	Bengochea	Me-LoP_columnar_Type1_3
Columnar	Optic Lobe	*Brachyura*	Bengochea	Me-LoP_columnar_Type1_5
Columnar	Optic Lobe	*Brachyura*	Bengochea	Me-LoP_columnar_Type2_3
Uniglomerular projection	Antennal lobe	*Drosophila melanogaster*	Jefferis	12070404c1
Uniglomerular projection	Antennal lobe	*Drosophila melanogaster*	Jefferis	CT12T2
Uniglomerular projection	Antennal lobe	*Drosophila melanogaster*	Jefferis	LHC6R
Shepherd's crook neuron	Mesencephalon	*Gallus gallus domesticus*	Marin	IMc
Shepherd's crook neuron	Mesencephalon	*Gallus gallus domesticus*	Marin	IPc
Shepherd's crook neuron	Mesencephalon	*Gallus gallus domesticus*	Marin	ShCr_Soma
Undefined	Neocortex	*Rattus*	Almeida	cm-ctx-e
Undefined	Neocortex	*Rattus*	Almeida	cm-ctx-f
Undefined	Neocortex	*Rattus*	Almeida	ctr-ctx-3-b

A table detailing the identity and sources of the axon reconstruction data extracted from the online database NeuroMorpho.Org. The standardized morphology files were used and manipulated based on the methods described in the main text in order to extract the radius and length scaling ratio distributions.

Table 5. NeuroMorpho.Org Reconstruction Data: Peripheral Nervous System Neurons

Cell Type	Region	Species	Archive Name	File Name
Dendritic arborization	Peripheral Nervous System	*Drosophila melanogaster*	Ye	021804-2b_ddaC-3-cd8_ch00
Dendritic arborization	Peripheral Nervous System	*Drosophila melanogaster*	Ascoli,Cox	11CL-IVxAnk2IR_ddaC
Dendritic arborization	Peripheral Nervous System	*Drosophila melanogaster*	Bellemer	36775-3
Sensory	Peripheral Nervous System	*Mus musculus*	Canavesi	control-contact-2
Sensory	Peripheral Nervous System	*Mus musculus*	Canavesi	control-noncontact-1
Sensory	Peripheral Nervous System	*Mus musculus*	Canavesi	diabetic-contact-4
Sensory	Peripheral Nervous System	*Mus musculus*	Yorek	image002
Sensory	Peripheral Nervous System	*Mus musculus*	Yorek	image008
Sensory	Peripheral Nervous System	*Mus musculus*	Yorek	image025_1
Somatic	Peripheral Nervous System	*Mus musculus*	Badea	Badea2012Fig6A-C-R
Somatic	Peripheral Nervous System	*Mus musculus*	Badea	Badea2012Fig6B
Somatic	Peripheral Nervous System	*Mus musculus*	Badea	Badea2012Fig6E-I-R
Touch receptor	Peripheral Nervous System	*Mus musculus*	Lumpkin	01-09-TD4
Touch receptor	Peripheral Nervous System	*Mus musculus*	Lumpkin	1-09-TD1-v3
Touch receptor	Peripheral Nervous System	*Mus musculus*	Lumpkin	1-09-TD4-v2

A table detailing the identity and sources of the Peripheral Nervous System neuron reconstruction data extracted from the online database NeuroMorpho.Org. The standardized morphology files were used and manipulated based on the methods described in the main text in order to extract the radius and length scaling ratio distributions.
